# Dysregulated TFR and TFH cells correlate with B‐cell differentiation and antibody production in autoimmune hepatitis

**DOI:** 10.1111/jcmm.14997

**Published:** 2020-03-06

**Authors:** Ma Liang, Zhang Liwen, Dai Juan, Zhuang Yun, Ding Yanbo, Chen Jianping

**Affiliations:** ^1^ Department of Gastroenterology The First People's Hospital of Changzhou The Third Affiliated Hospital of Soochow University Changzhou China; ^2^ Department of Pediatrics The Second People's Hospital of Changzhou Affiliate Hospital of NanJing medical University Changzhou China

**Keywords:** AIH, EAH, TFH cells, TFR cells

## Abstract

Follicular helper T (TFH) cell provides germinal centre (GC) B cell with critical signals for autoantibody production in the immunopathogenesis and progression of autoimmune hepatitis (AIH). However, the immunoregulatory functions of follicular regulatory T (TFR) cell in AIH are still unclear. The numbers of circulating TFR/TFH cells were measured in AIH patients. Moreover, we established experimental autoimmune hepatitis (EAH) model to examine the function of TFR cells on B‐cell differentiation and autoantibody production in vivo and vitro. AIH patients had significantly increased numbers of TFH cells and decreased numbers of TFR cells as well as imbalanced TFR/TFH‐type cytokines (IL‐10, TGF‐β1 and IL‐21) compared with healthy controls (HCs). In addition, TFR cell numbers negatively correlated with TFH cell numbers. Also, serum hypergammaglobulinaemia (IgG and IgM) concentration negatively correlated the levels of serum IL‐21, but positively correlated with the levels of serum IL‐10 in AIH patients. Furthermore, in comparison with control group, significantly higher frequencies of spleen TFR cells but lower frequencies of spleen TFH cells were detected in the EAH group. Further analysis found that TFR cells simultaneously express the phenotypic characteristics of Treg and TFH cells, but exercise as negative regulators of autoantibody production in vitro culture. Our findings demonstrated that dysregulated between TFR and TFH cells might cause excessive production of autoantibodies and destruction of the immune homeostasis, leading to the immunopathological process in AIH.

## 1 INTRODUCTION

Autoimmune hepatitis (AIH) is a severe progressive chronic autoimmune disease. AIH is characterized by high levels of hypergammaglobulinaemia and hepatic inflammatory infiltrates, leading to potential cirrhosis.^1^ While environmental parameters are implicated,^2^, ^3^ immunological dysfunction plays a crucial role in the pathogenesis of AIH.^4^, ^5^, ^6^, ^7^ However, the exact mechanisms of autoimmune responses remain unclear.

It is generally believed that abnormal selection of high‐affinity autoantibody‐producing plasma cells in germinal centre (GC) plays a central role of AIH.^8^ We previously characterized hyperactive B‐cell immunity in AIH patients and found that original B cells can differentiate into CD27^+^ memory and CD138^+^ plasma cells that produce autoantibodies, such as anti‐nuclear antibodies.^4^ This differentiation process of B cells requires the help of TFH cells,^4^, ^9^, ^10^, ^11^ which is one of the important regulators of humoural immune responses. TFH cells express high levels of inducible T‐cell co‐stimulator (ICOS), programmed death 1(PD‐1) and B‐cell lymphoma 6, a master transcriptional activator of TFH differentiation.^9^, ^10^, ^11^ In the GC, TFH cell provides signals for B‐cell survival and differentiation via the coreceptor ICOS and CD40L expression.^12^, ^13^ Besides direct B‐TFH contact, TFH cell also promotes the generation of Abs with high affinity through soluble mediators like interleukin‐4 (IL‐4) and IL‐21.^9^, ^10^, ^11^ Uncontrolled accumulation of TFH cell might activate autoreactive B cells to produce excessive autoantibodies that cause autoimmune responses.^14^, ^15^ Our previous study also confirmed that TFH cell has been detected in the peripheral circulation, and the excessive activation of TFH cells accelerated immunopathological process of AIH.^4^ In addition, circulating activated TFH cells are associated with hypergammaglobulinaemia.^4^ Hence, the exact regulation of TFH cell‐mediated antibody production is essential for preventing AIH.

More notably, a newly identified T‐cell subset, termed follicular regulatory T(TFR) cell, propose themselves as ideal candidates for preventing emergence of autoreactive B cells and regulating the normal GC response.^16^, ^17^ TFR cell combining phenotypic characteristics with conventional Foxp3^+^Tregs and TFH cell yet are not identical to both.^16^, ^17^, ^18^ Similar to TFH cell, TFR cell migrates to the GC by expressing CXCR5, which is a chemokine receptor for CXCL13. Furthermore, both subsets commonly express ICOS, PD‐1 and Bcl6.^16^, ^17^ Unlike TFH cell, which normally differentiate from naive CD4^+^T cell precursors, TFR cell originated from thymic Foxp3 + Treg precursors.^17^ Therefore, in comparison with TFH cell, TFR cell has unique characteristics by the expressions of Treg‐related molecules.^18^ Research confirmed that TFR cell simultaneously expresses the Treg master regulator Foxp3 and other Treg‐related molecules such as CD25 and cytotoxic T‐lymphocyte–associated protein 4 (CTLA‐4).^16^, ^17^, ^18^ Several studies have showed that abnormal TFR cells activity might cause the destruction of immune tolerance and thereby lead to the immunopathogenesis of autoimmune diseases.^19^, ^20^ Additionally, like TFH cell, TFR cells are also present in the peripheral circulation, the levels of which are directly correlated with a variety of human immunological disorders, such as primary biliary cholangitis (PBC) and multiple sclerosis(MS).^21^, ^22^ However, to the best of our knowledge, there are no reports of circulating TFR cells in patients with newly diagnosed AIH. Furthermore, the potential mechanisms underlying the function of TFR cells remain to be elucidated.

In the current study, our data indicated that imbalance of TFR/TFH ratio involves in the pathogenesis of AIH and provides new insights into how TFR cell regulates humoural immune responses and potential therapeutic targets for the development of novel therapies for AIH.

## 2 MATERIAL AND METHODS

### 2.1 Participants

We recruited 32 AIH patients at the inpatient clinic of the Department of Gastroenterology, the First People's Hospital of Changzhou. All patients were diagnosed in an active disease state, as defined by an alanine aminotransferase (ALT) value or aspartate aminotransferase (AST) value >50 U/mL, according to the international criteria for the definitive diagnosis of AIH type I.^23^ Another 20 age‐, gender‐ and ethnicity‐matched healthy controls (HCs), who had no history of any chronic inflammatory disease, were recruited from the Department of Medical Examination Center of the First People's Hospital of Changzhou during the same period. An informed consent was written from individual participants, and the experimental protocol was approved by the Ethical Committee of the First People's Hospital of Changzhou.

### 2.2 Clinical index assay

The concentrations of serum alanine transaminase (ALT) and aspartate aminotransferase (AST), anti‐nuclear and smooth muscle antibody (ANA/SMA), IgG, IgM and IgA were determined according to the manufacturers' instruction (Beckman Coulter).

### 2.3 Animals

SPF‐class female C57BL/6 mice, aged 6‐8 weeks, body weights 18‐20 g, were purchased from Nanjing Experimental Animal Center (Jiangsu, China). Above all, all animal experiments were carried out in accord with the Guide for the Care and Use of Laboratory Animals, and were approved by the Laboratory Animal Ethics Committee of the First People's Hospital of Changzhou.

### 2.4 Establishment of experimental autoimmune hepatitis (EAH) model

Liver antigens were always prepared freshly as described previously from C57BL/6 female mice after perfusion of livers with phosphate‐buffered saline (PBS). Livers were homogenized on ice, and nuclei and remaining intact cells were centrifuged at 150 *g* for 10 minutes. Subsequently, the supernatants were centrifuged for 1 hour at 100 000 *g*, and the remaining supernatants used for immunization (called S100). Induction of experimental autoimmune hepatitis (EAH) was achieved by intraperitoneal injection of the mice with freshly prepared S‐100 antigen at a dose of 0.5‐2 mg/mL in 0.5 mL PBS that had been emulsified in an equal volume of complete Freund's adjuvant (CFA) on day 0. A booster dose was given on day 7 as well.^5^, ^6^, ^20^ Disease severity was assessed histologically on day 28 when the peak of disease activity was observed. Disease severity was graded on a scale of 0‐3 by a researcher who was blinded to the sample identity, as follows: grade 0, none; grade 1, mild‐scattered foci of lobular‐infiltrating lymphocytes; grade 2, moderate‐numerous foci of lobular‐infiltrating lymphocytes; grade 3, severe‐extensive pan‐lobular‐infiltrating lymphocytes.^5^

### 2.5 Flow cytometric analysis

Peripheral blood mononuclear cells (PBMCs) and spleen mononuclear cell (SMNCs) were isolated from AIH patients and EAH mice by density gradient centrifugation according to the manufacturer's instruction. To detect different T‐cell subsets, PBMCs or SMNCs (1 × 10^6^/tube) were stained with BV510‐anti‐CD4 (0.2 mg/mL), PerCP‐Cy5.5‐anti‐CXCR5 (0.1 mg/mL), PE‐Cy7‐anti‐CD25 (0.05 mg/mL), BV421‐anti‐ICOS (0.05 mg/mL), FITC‐anti‐PD‐1 (0.05 mg/mL), APC‐anti‐CTLA‐4(0.2 mg/mL) (BD Biosciences) in the dark at 4°C for 30 minutes, fixed, permeabilized using transcription factor Fix/Perm buffer, and stained with antibodies for intracellular proteins including PE‐anti‐FoxP3(0.1 mg/mL), PE‐CF594‐anti‐IL‐10(0.05 mg/mL) and Alexa Fluor 647‐anti‐IL‐21(0.1 mg/mL) (BD Biosciences). After washing with transcription factor Perm/wash buffer (BD Biosciences), the numbers of each phenotype of TFR and TFH cells in AIH patients and the frequency of GITR^+^TFR and GITR^‐^TFH in EAH mice were characterized by flow cytometry analysis.

### 2.6 Enzyme‐linked Immunosorbent assay for IL‐10, TGF‐β1 and IL‐21

The concentrations of serum/supernatant IL‐10, TGF‐β1 and IL‐21 in individual subjects were determined by ELISA using commercially available IL‐10, TGF‐β1 and IL‐21 ELISA kits (R&D system) according to the manufacturer's instructions.

### 2.7 Cell culture in vitro

We pre‐purified CD4^+^CXCR5^+^GITR^‐^TFH and CD4^+^CXCR5^+^GITR^+^TFR cell subsets from spleen cells of wild‐type mice and B cells from spleen cells of EAH mice (grade =3) by immunomagnetic beads. The purity of TFR/TFH and B cells was up to 97%. To a U‐bottom 96‐well culture dish, add 50 × 10^3^ sorted B cells were stimulated with anti‐CD3 (2μg/ml) and anti‐IgM (5 μg/mL) (BD Biosciences) (diluted 1:200 in Perm/Wash), and then co‐cultured with 1 × 105 sorted TFH cells, in the absence or presence of 15 × 10^3^ sorted TFR cells in 10% FCS RPMI‐1640 (Hyclone) for 6 days at 37°C. Experimental groups should include B + TFH, B + TFR, B + TFR+anti‐CTLA4 Abs, B + TFH+TFR cells and B cells alone. For B‐cell functional assay, the concentrations of supernatants total IgG from the cocultures were measured by ELISA. For analysis of B‐cell differentiation, the frequency of CD138 + plasma and CD27 + memory cells was determined by flow cytometry.

### 2.8 Statistical analysis

The difference between two independent groups was analysed by Fisher's exact test or the Kruskal‐Wallis nonparametric test using the SPSS 18.0 software(SPSS, Inc). The relationship between variables was evaluated using the Spearman rank correlation test. A two‐sided *P* value <.05 was considered statistically significant.

## 3 RESULTS

### 3.1 Patient characteristics

The clinical and sociodemographic characteristics of recruited subjects were described in Table 1. In comparison to HCs, patients had significantly higher concentrations of serum liver enzymes (ALT/AST/γ‐GT and ALP), and higher the levels of serum immunoglobulin (IgG, IgM and IgA). Furthermore, the majority of AIH patients were seropositive for anti‐ANAs and anti‐SMA antibodies. Consistently, all AIH patients displayed active disease and hypergammaglobulinaemia.

**Table 1:** Clinical characteristics of AIH patients and Healthy controls

Parameters	AIH	HC
NO	32	20
Age (years)	48 (37‐76)	51 (41‐74)
Gender: female/male	24/8	14/6
ALT (U/L)	125.9 ± 108.3*	27.2 ± 8.2
AST (U/L)	101.1 ± 53.7*	22.7 ± 5.7
γ‐GT (U/L)	89.1 ± 30.3*	25.1 ± 7.4
ALP (U/L)	133.4 ± 37.1*	89.5 ± 23.6
Bilirubin (umol/L)	12.5 ± 8.1*	10.8 ± 6.8
Albumin (g/L)	23.8 ± 5.7	25.3 ± 4.8
PT‐INR	1.0 ± 0.9	1.1 ± 0.6
Anti‐ANA (+)	23/32 (71.8%)*	0/20 (0%)
Anti‐ANA titre	1:640 (1:80‐1:10 000)	‐
Anti‐SMA (+)	2/32 (6.25%)	0/20 (0%)
Anti‐SMA titre	1:1000 (1:160‐1:3200)	‐
IgG (g/L)	15.9 ± 3.7*	7.8 ± 2.3
IgM (g/L)	6.9 ± 1.9*	2.64 ± 0.87
IgA (g/L)	4.07 ± 2.3*	1.6 ± 1.1
WBC (*10 9/L)	7.93 (5.6‐11.2)*	5.08 (3.9‐9.2)

Note

Data shown are real case number or mean ± SD.

Normal values: ALP, alkaline phosphatase: 45‐125 μ/L; albumin: 35‐53 g/L; ANA, anti‐nuclear antibody: <1:80; SMA, anti‐mitochondrial antibodies: <1:80; HC, healthy control; AIH, autoimmune hepatitis; ALT, alanine aminotransferase: 5‐40 U/L; AST, aspartate transaminase: 5‐40 U/L; γ‐GT, gamma‐glutamyl transferase: 10‐60 μ/L; IgA:0.4‐2.3 g/L; bilirubin: 3.4‐20.5 umol/L; IgM: 0.7‐4.6 g/L; IgG: 7‐16 g/L.

* *P* < .05 vs HC.

### 3.2 Decreased numbers of circulating TFR cells and increased numbers of circulating TFH cells in AIH patients

**Figure 1:**
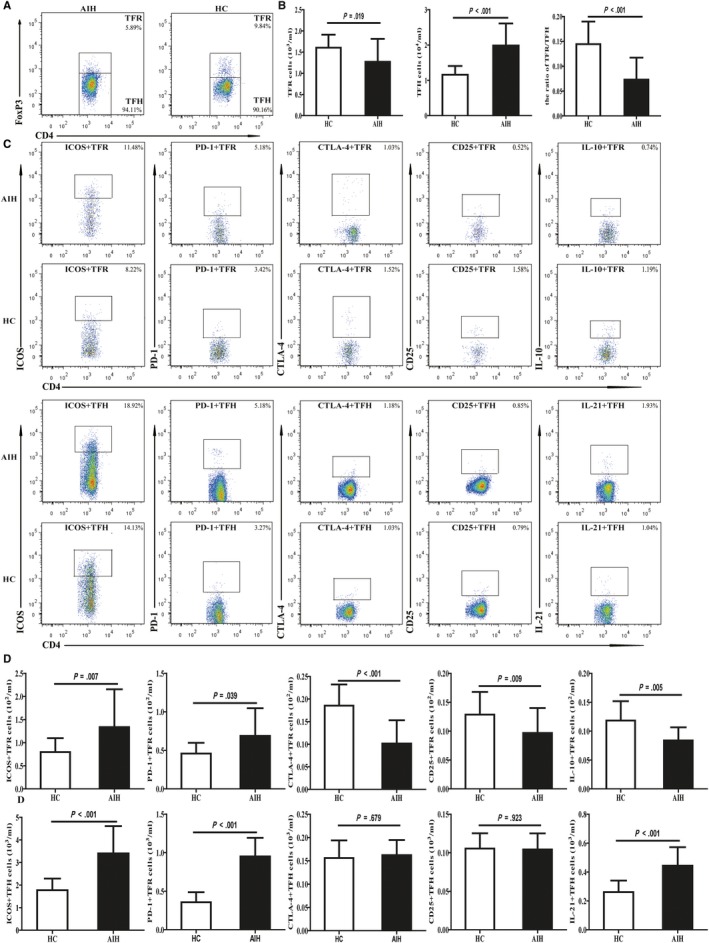
Flow cytometry analysis of the numbers of circulating TFR and TFH cells in AIH patients and HCs. PBMCs 5*10^5^/tube were isolated from individual subjects and were stained in duplicate with anti‐CD4, anti‐CXCR5, anti‐ICOS, anti‐PD‐1, anti‐CTLA‐4, anti‐CD25 and intracellular anti‐Foxp3, anti‐IL‐21 or IL‐10, respectively. The cells were characterized by flow cytometry analysis by gating initially on living lymphocytes, and then on CD4^+^CXCR5^+^Foxp3^+^TFR and CD4^+^CXCR5^+^Foxp3^‐^TFH cells. Subsequently, the numbers of different subsets of TFR and TFH cells were calculated, according to the total numbers of PBMCs, the frequency of TFR and TFH cells. A, Flow cytometry analysis of TFR and TFH cells; B, the numbers of CD4^+^CXCR5^+^Foxp3^+^TFR, CD4^+^CXCR5^+^Foxp3^‐^TFH cells; and TFR/TFH ratio; C, flow cytometry analysis of different subsets of TFR and TFH cell; D, the numbers of ICOS^+^, PD‐1^+^, CTLA‐4^+^, CD25^+^ and IL‐10^+^ TFR and TFH cells. Data shown are representative FACS charts or the mean numbers of each type of cells per mL of peripheral blood in individual subjects from two separate experiments. The horizontal lines indicate the median values for each group

Follicular helper T cell originates from peripheral Foxp3‐T cells, in contrast to TFR cell, which originate from thymic‐derived Foxp3 + T cell.^18^ According to the expression patterns of FoxP3, peripheral blood CD4^+^CXCR5^+^T cells were divided into circulating TFR and TFH cell subsets. The gating strategy for flow cytometric analysis of TFR (CD4^+^CXCR5^+^FoxP3^+^) and TFH (CD4^+^CXCR5^+^FoxP3^‐^) cells was shown in Figure 1A. In contrast to HCs, TFR cells expression and TFR/TFH ratio declined, but TFH cells expression increased in AIH patients (Figure 1B). Hence, imbalanced between TFR and TFH cells may be associated with the pathogenesis of AIH.

### 3.3 Circulating TFR express markers of both TFH and Treg differentiation in AIH patients

In the GC, TFR cell shares phenotypic, ontological and functional characteristics with conventional Tregs and TFH cell.^16^, ^17^, ^18^ This led us to question whether circulating follicular cells are phenotypically similar to those derived from tonsils using both follicular (ICOS and PD‐1) and regulatory (CTLA‐4 and CD25) markers (Figure 1C, 1). We found that circulating TFH cells express higher levels of PD‐1 and ICOS, and similar levels of CTLA‐4 and CD25 in AIH patients compared with HCs. As a comparison, circulating TFR cells express higher levels of PD‐1 and ICOS, and lower levels of CTLA‐4 and CD25 in AIH patients. In addition, we also detect a lower numbers of IL‐10^+^TFR cells and a greater numbers of IL‐21^+^TFH cells in AIH patients compared to that in the HC (Figure 1C, 1). These data suggest that TFR cell that simultaneously expresses the phenotypic characteristics of TFH and Tregs played a negative regulatory role in the pathogenesis of AIH.

### 3.4 TFR/TFH‐Related Cytokines in AIH Patients

**Figure 2:**
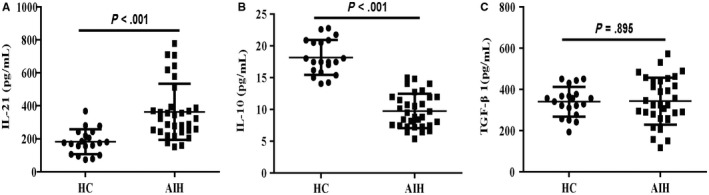
Serum levels of TFR/TFH‐type cytokines in AIH patients and HCs. Serum levels of A, IL‐21; B, IL‐10; C, TGF‐β1in AIH patients and HCs. Data shown are the mean levels of each serum cytokine in individual subjects from two separate experiments. The horizontal lines indicate the mean values for the different groups

Follicular regulatory T cell secretes IL‐10 and TGF‐β1, which are functionally important for the regulatory/suppressive activities of TFR cell,^19^, ^20^ whereas TFH cell produces IL‐21, which is critical for GC formation as well as TFH cell generation.^9^, ^10^, ^11^ We further tested the levels of these serum cytokines and found a lower concentrations of serum IL‐10 and a higher concentrations of serum IL‐21 in the patients compared with those in HCs. However, the serum levels of TGF‐β1 showed no significant difference between HCs and AIH patients (Figure 2). These data clearly indicated that elevated levels of serum IL‐21 and reduced levels of serum IL‐10 may be associated with the development of AIH.

### 3.5 Circulating TFR cells are inversely associated with TFH cells in AIH patients

**Figure 3:**
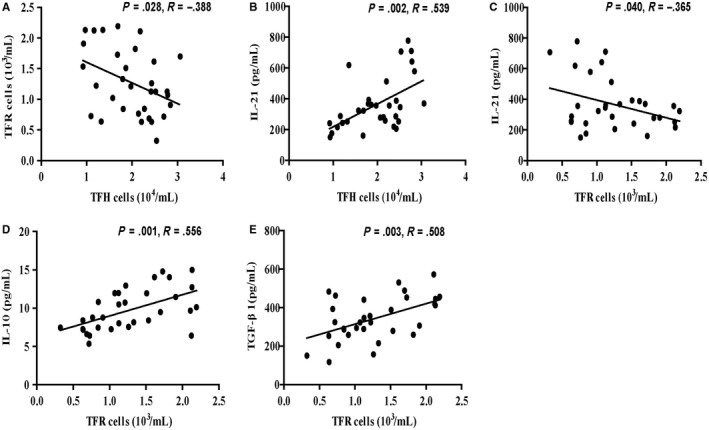
Correlation among the numbers of different subsets of TFR and TFH cells and the levels of serum IL‐21, IL‐10 and TGF‐β1 in AIH patients. A, Correlation between the numbers of TFR cells and the numbers of TFH cells in AIH patients; B, correlation between the numbers of TFH cells and serum levels of IL‐21 in AIH patients; C, correlation between the numbers of TFR cells and serum levels of IL‐21 in AIH patients; D, correlation between the numbers of TFR cells and serum levels of IL‐10 in AIH patients; E, correlation between the numbers of TFR cells and serum levels of TGF‐β1 in AIH patients

To further analyse the relationship among the numbers of TFR/TFH cells and the levels of serum TFR/TFH‐related cytokines, we next performed a set of correlation analyses and found that the numbers of circulating TFR cells in AIH patients are positively associated with the levels of serum IL‐10 (Figure 3D) and TGF‐β1 (Figure 3E), but negatively correlated with the numbers of circulating TFH cells (Figure 3A) as well as the levels of serum IL‐21 (Figure 3C). In addition, the numbers of circulating TFH cells are associated with the concentrations of serum IL‐21 in those patients (Figure 3B). These data suggest that TFR cell negatively affects the formation of TFH cell and the production of IL‐21 during the development of AIH.

### 3.6 Correlations of TFR/TFH‐Related cytokines with clinical parameters in AIH patients

**Figure 4:**
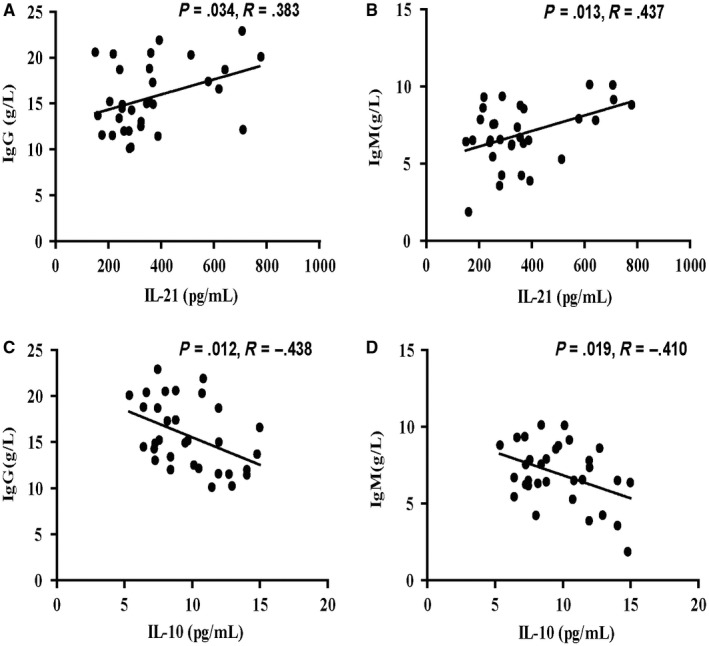
Correlation between serum levels of TFR/TFH‐type cytokines and the values of clinical parameters in AIH patients. A‐B, Correlation between serum levels of IL‐21 and titre IgG/IgM in AIH patients; C‐D, correlation between serum levels of IL‐10 and titre IgG/IgM in AIH patients

Next, we further determined the associations among the levels of serum IL‐21/IL‐10 and clinical parameters of AIH patients. We observed that the serum IL‐21 levels were associated positively with the concentrations of serum IgG and IgM (Figure 4A‐B), but the serum IL‐10 levels were inversely associated with the concentration of serum IgG and IgM (Figure 4C‐D). However, our data did not show any significant correlations between the levels of TFR/TFH‐type cytokines and other parameters tested in those patients. Overall, the concentrations of serum TFR/TFH‐related cytokines may be valuable markers for the evaluation of disease activity in AIH patients.

### 3.7 Statistical decrease in TFR cells and increase in TFH cells in EAH mice

**Figure 5:**
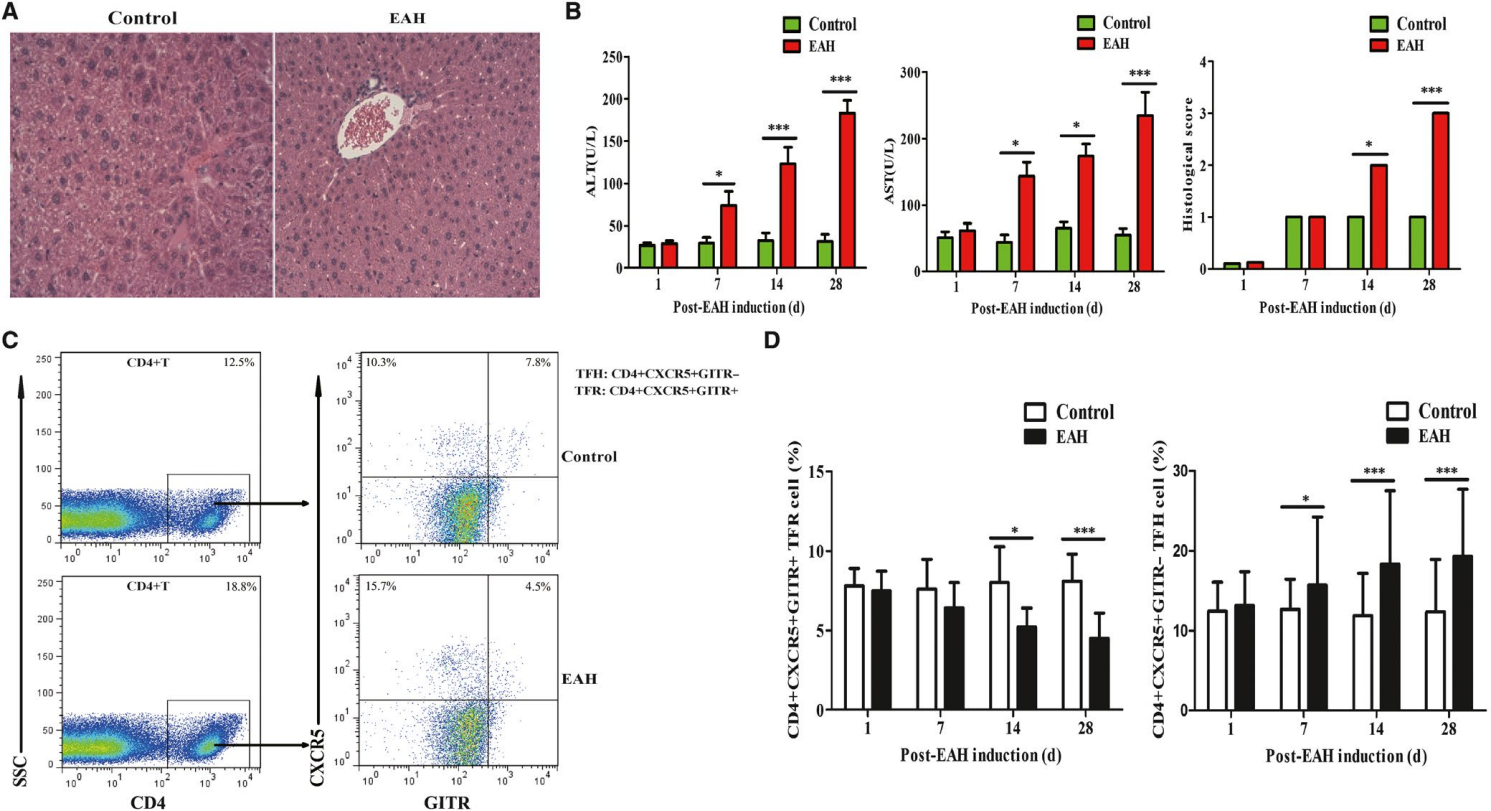
Flow cytometry of TFR/TFH cells in SMNCs in EAH mice. Ten mice (five from the EAH group and five from the control group) were killed at each time‐point (1, 7, 14 and 28 days). A, Representative histological picture of liver lesions in animals after standard induction of EAH and controls on the 28th day (magnification, 200×); B, serum ALT/AST levels progressively up‐regulated from 1 to 28 days and histological score of liver lesions in mice after standard induction of EAH; C, flow cytometry analysis of TFR and TFH cells from the spleen on the 14th day; D, percentages of TFR and TFH cells at each time‐point from EAH and control mice were analysed by FACS. The horizontal lines indicate the mean values of the different groups. *<0.05; ***<0.01

To assess the effects of follicular cells in vivo, our previous studies had successfully established the autoimmune hepatitis model of EAH mice (Figure 5A, 5).^5^, ^6^ Considering that GITR by TFR cells has enabled sorting strategies to highly purify TFH and TFR cells,^16^ we further analysed the percentages of CD4^+^CXCR5^+^GITR^+^TFR and CD4^+^CXCR5^+^GITR^‐^TFH cells in EAH mice (Figure 5C). We observed that CD4^+^CXCR5^+^GITR^+^TFR cells in the experimental group significantly decreased from 14th to 28th days of post‐EAH induction. On the other hand, CD4^+^CXCR5^+^GITR^‐^TFH cells in the experimental group gradually increased from 7th to 28th days of post‐EAH induction compared with control group (Figure 5D). These results indicated that the imbalance of TFR‐to‐TFH ratios might favour the pathogenesis of EAH.

### 3.8 TFR regulates B‐cell subset differentiation and immunoglobulin production in vitro

**Figure 6:**
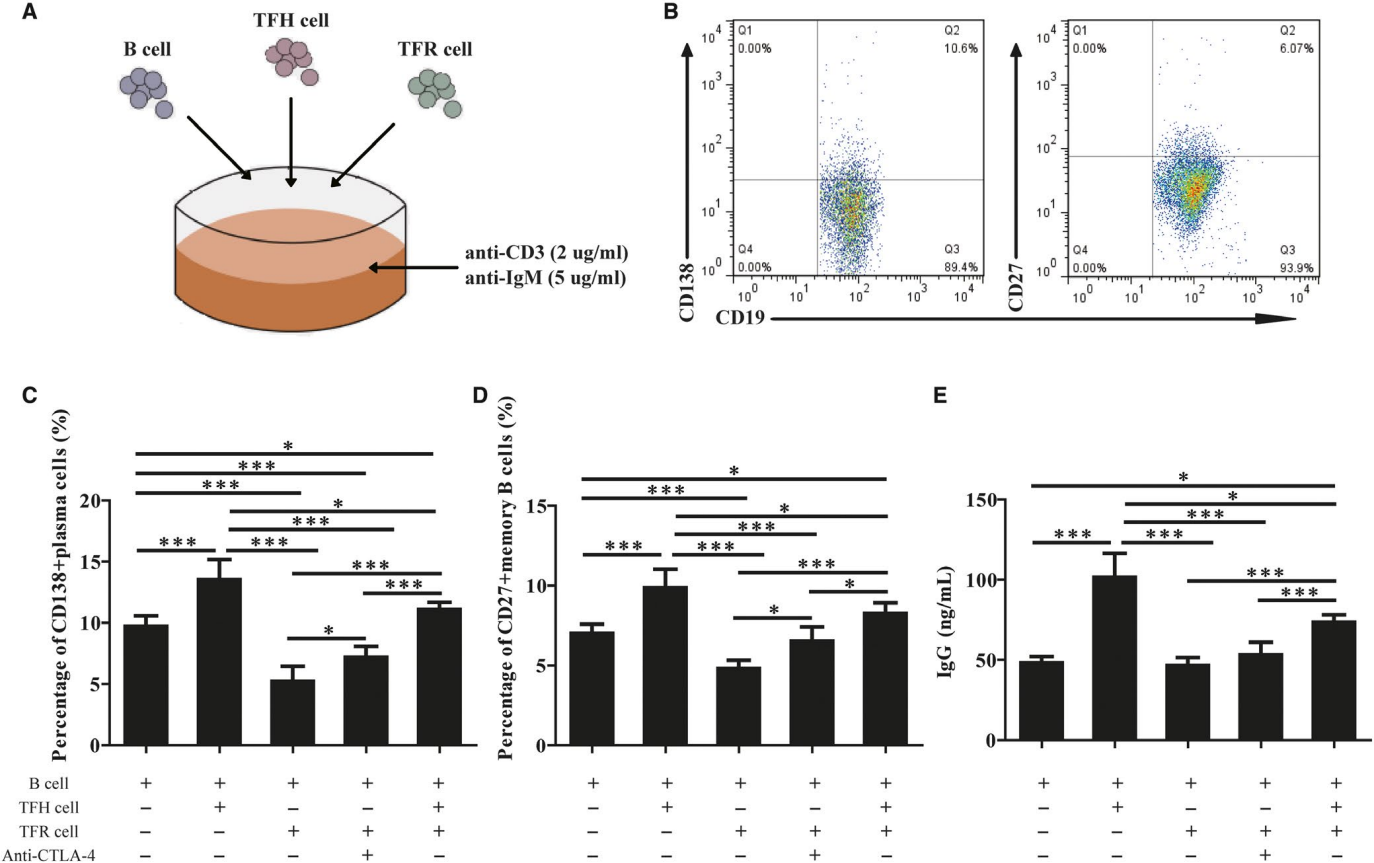
Follicular regulatory T cell suppresses B‐cell differentiation and antibody production in vitro. B cells obtained from the spleen of EAH mice (Grade = 3), incubated with wild‐type TFH cells in the presence or absence of TFR cells together in to cultures along with or without anti‐CTLA‐4 Abs. A, Cell culture plating schematic. TFR, TFH and B cells were sorted according to protocol and added together into cultures along with anti‐CD3 and anti‐IgM; B, flow cytometry analysis of CD138^+^ plasma cells and CD27^+^ memory cells; (C‐D) percentages of CD138^+^ plasma cells and CD27^+^ memory cells in different coculture group; E, titres of IgG in supernatants of different coculture group. The horizontal lines indicate the mean values of the different groups. *<0.05; ***<0.0

To identify potential mechanisms by which TFH cell and B cell are regulated by TFR cell^24^, we co‐cultured spleen B cells from EAH mice with TFH cells from wild‐type mice for 7 days in the absence and/or presence of TFR cells from wild‐type mice, anti‐CTLA antibodies (Figure 6A). Next, we characterized the expression of CD138 and CD27 on CD19 + B cells from cultures by flow cytometry analysis (Figure 6B). As shown in the Figure 1C‐E, we observed higher percentage of memory B and plasma cells and higher levels of IgG when B cell was co‐cultured with TFH cells. However, after TFR cells were added to the wells together with TFH cells, the percentages of memory B and plasma cells as well as the production of IgG were significantly dramatically decreased. On the other hand, when anti‐CTLA antibodies were added, the percentages of these cells and the production of IgG were slightly increased (Figure 6B‐E). Together, these data suggest that TFR cell can suppress B‐cell proliferation and antibody production in vitro.

## 4 DISCUSSION

Although TFR cells are known to be important regulators of autoimmune responses in human,^19^, ^20^, ^21^, ^22^ the precise role of TFR cell in the pathogenic processes of AIH remains unclear. This study showed sufficient evidence that dysregulated between TFR and TFH cells contribute to the immunopathological process in AIH. We observed greater numbers of TFH cells in EAH mice and AIH patients, which are consistent with ther previous finding.^4^ Further, our study showed decreased numbers of TFR cells in AIH patients and EAH mice. More importantly, the hypothesis that TFR cell plays an immune regulatory role in vivo is supported by the current study that TFR cells were inversely associated with TFH cells and serum IL‐21 levels, but positively associated with reduced serum IL‐10 and TGF‐β1. In addition, elevated serum IL‐21 levels are positively associated with immunoglobulin levels, whereas decreased serum IL‐10 levels are inversely associated with immunoglobulin levels, which might regulate immunoglobulin production.^25^, ^26^ Taken together, our data revealed the pathogenic significance of altered TFR/TFH cell balance in AIH.

Next, we confirmed that circulating TFR cells show distinct yet overlapping phenotype as compared with TFH and Treg cell based on a combination of flow cytometric analyses. The observation that TFR cell expresses proteins typically expressed by TFH cell, such as ICOS and PD‐1, as well as proteins typically expressed by Tregs, like CD25 and CTLA4, is consistent with studies showing that TFR cells are thymic‐derived Treg that migrate into the follicle in a TFH cell‐dependent manner, and up‐regulate CXCR5 and PD‐1 in a manner similar to plasma cell.^16^, ^17^, ^18^ Like TFH cell, we found significantly higher levels of PD‐1^+^TFR and ICOS^+^TFR cells in AIH patients. These data suggested that activated TFR cells might provide negative signals to repress aberrant B‐cell activation and differentiation. Furthermore, we found a diminished CTLA‐4 expression on TFR cells. As a inhibitory molecule, CTLA‐4 is an important modifier for maintaining immunological self‐tolerance and immune homeostasis.^27^, ^28^ Recent studies reported that depletion of CTLA‐4 on TFR cells might result in defective suppression of autoimmune responses.^29^, ^30^ This findings are consistent with our observation that decreased CTLA‐4 might augment cell‐mediated immune responses and antibody production. Collectively, our findings indicated that the decreased CTLA‐4 and increased PD‐1/ICOS on TFR cells might be involved in AIH pathogenic process by regulating B‐cell response.

Although TFR cell potently controls autoantibody secretion, the precise mechanisms by which TFR cell exerts these immunoregulatory functions need to be explored further. Consistent with previous published reports, our findings confirmed that activated TFH cells can induce B‐cell activation and differentiate into memory B and plasma cell, which might aid in antibody production.^4^, ^9^, ^10^, ^11^, ^12^, ^13^ However, in vitro culture experiments have shown that TFR cells can repress the differentiation of B cell and the secretion of IgG antibodies. One possible underlying mechanism of TFR cell suppression is attributed to the expression of the coreceptor CTLA, which might exert negative regulatory effects.^28^, ^29^, ^30^ Another reason is due to the secretion of inhibitory cytokines such as TGF‐β1 or IL‐10.^16^, ^17^, ^18^, ^31^, ^32^ Related studies have reported that IL‐10 modulates the secretion of antibody, and TGF‐β1 secreted by TFR cells can inhibit TFH function.^19^, ^20^, ^31^, ^32^ This is a reasonable explanation for our experimental observation that when TFR cells were added co‐cultured with TFH cell and B cells, B‐cell differentiation and production of IgG were reduced in culture supernatants. In summary, our study indicated that TFR cell might physically disrupt TFH cell‐ and B‐cell–linked recognition during suppression.

Taken together, the presented data described the subset phenotypes of circulating TFR and TFH cells and their relationships with clinical parameters in AIH patients. In addition, our study also showed that dysregulated between TFR and TFH cells might contribute to the immunopathological process in AIH. In vitro culture experiments demonstrated that TFR cells could inhibit the secretion of autoantibody by indirectly inhibiting the activation of TFH cells, or directly regulating the differentiation of B cells via the coreceptor CTLA‐4. We recognized the limitations of this study including a relatively small sample size and the lack of vivo functional study of TFR cells. Therefore, further studies of these findings in a larger populations are warranted.

## CONFLICTS OF INTEREST

The authors declare that they have no financial or commercial conflicts.

## AUTHORS' CONTRIBUTIONS

Ma Liang, Zhang Liwen and Dai Juan contributed equally to this work. Chen Jianping and Zhuang Yun designed the experiments and analysed data. Ma Liang wrote the main manuscript text. Ma Liang, Zhang Liwen and Dai Juan performed the experiments and prepared the figures. Dan Juan and Ding Yanbo provided samples. Chen Jianping provided conceptual and technical advices; and all authors reviewed the manuscript.

## Data Availability

All data included in this study are available upon request by contact with the corresponding author.
